# Soil Microbiome Predator Diversity Outperforms Nitrogen Addition in Boosting Plant Biomass via Bacterial Community Shifts

**DOI:** 10.1111/gcb.71019

**Published:** 2026-08-12

**Authors:** Alejandro Berlinches de Gea, Joshua Both, Nina Haas, Rutger A. Wilschut, Florian Wichern, Stefan Geisen

**Affiliations:** ^1^ Laboratory of Nematology Wageningen University & Research Wageningen the Netherlands; ^2^ Fruit Production Extension, Department of Plants and Plant Products Agroscope Wädenswil Switzerland; ^3^ Ecology, Department of Biology University of Konstanz Konstanz Germany; ^4^ Soil Science and Plant Nutrition, Sustainable Food Systems Research Centre Rhine‐Waal University of Applied Sciences Kleve Germany

## Abstract

Nitrogen (N) is crucial for plant growth, but its overuse harms biodiversity. Increasing soil biodiversity might provide the means to reduce N inputs, but experimental evidence for this paradigm shift is limited. Using microbiome predators (protists and nematodes) that shape microbiome composition and participate in N cycling, we examined how interactions between their diversity and N addition affect *Cannabis sativa* growth. The addition of microbiome predators increased plant biomass by up to 53%, irrespective of diversity level, with effects reaching up to 60% under higher microbiome predator diversity. This biomass increase was primarily associated with changes in bacterial community composition and enriching functions related to carbon and N cycling. In contrast, N input played a greater role in determining plant and soil nutrient content. These findings suggest that microbiome predators determine plant biomass in a diversity and N‐specific manner, showing the pivotal role of soil biodiversity in enhancing plant performance and serving as promising tools to mitigate N inputs.

## Graphical abstract

Soil biodiversity may help reduce dependence on nitrogen fertilization, but experimental evidence remains limited. We tested how microbiome predator diversity (protists and nematodes) and nitrogen addition influence *Cannabis sativa* performance. Increasing microbiome predator diversity enhanced plant biomass by up to 60%, exceeding the effects of nitrogen addition. Structural equation modelling revealed that predator diversity primarily affected plant growth through shifts in bacterial community composition, whereas nitrogen addition mainly influenced soil and plant nutrient content. These findings highlight microbiome predator diversity as a key driver of plant productivity.

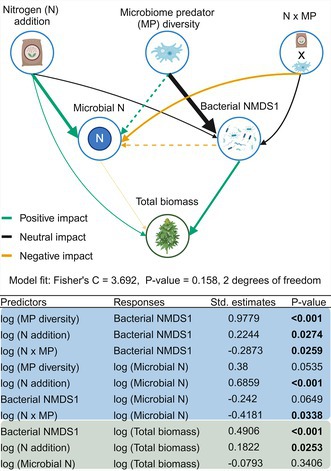

## 1 Introduction

Inorganic nitrogen (N) fertilization was part of the green revolution and ever since has provided the basis to feed the growing human population (Tscharntke et al. 2012). However, crops in agricultural systems take up only 60% of added N and N use efficiency declines with agricultural intensification (Sainju et al. 2019; Zhang et al. 2017). The remaining N leaches out of agricultural systems and negatively affects the environment through the accumulation of nitrate in groundwater, soil acidification, and increased greenhouse gas emissions (Sainju et al. 2019; Zhao et al. 2020). Excess N also reduces biodiversity, such as that of plants and insects (Emmerson et al. 2016; Humbert et al. 2016), by favoring opportunistic ruderal and pest species that thrive under increased N availability (Lekberg et al. 2021).

The loss of biodiversity, as explained by the biodiversity and ecosystem functioning (BEF (Tilman et al. 2014)) concept, has far‐reaching consequences: a reduced biodiversity (here: species richness) is commonly associated with a loss in ecosystem functions. For instance, in a global study, Hautier et al. (2018) showed that higher‐diverse grassland communities exhibited a higher multifunctionality compared to lower‐diverse grassland communities. While mainly shown for plants, this BEF relationship also has been proven for different organismal groups (Cardinale et al. 2012; Hu et al. 2016; Isbell et al. 2015) and is explained by the increasing complementarity of functions in the multidimensional niche space (Brooker et al. 2021; Hautier et al. 2018). However, the soil BEF (sBEF) concept so far showed relationships to range from positive to negative, which are determined by soil biophysiochemical properties such as N content, pH, or water percentage (Berlinches de Gea, Hautier, and Geisen 2023).

The little attention given to sBEF compared to BEF contradicts the fact that soils host ~59% of the total biodiversity on our planet (Anthony et al. 2023), determining essential ecosystem functions like carbon (C) and N cycling or plant growth (Bardgett and van der Putten 2014; Crowther et al. 2024). Among these ecosystem functions, bacterial‐driven N cycling is particularly important (Banerjee and van der Heijden 2023; Geisen et al. 2019; Singh et al. 2023), especially in N‐limited soils (Dong et al. 2024; Fierer 2017), while in agricultural soils plant's dependency on naturally occurring N‐increasing processes has been removed by fertilization. The role of bacteria in ecosystem functioning is affected by microbiome predators, which are key drivers of bacterial community composition. Microbial protist and metazoan nematodes (both together termed microbiome predators) predominantly feed on the classical microbiome, being bacteria and fungi. These microbiome predators release nutrients immobilized in bacteria and fungi through predation, enhancing N availability via the microbial loop (Bardgett and Chan 1999; Bonkowski 2004; Clarholm 1985). By selective feeding, microbiome predators shift bacterial communities (Hu et al. 2023), influencing functions, like N and C cycling (Bonkowski 2004; Gao et al. 2019). However, most studies on species‐specific prey selection focus on single model microbiome predator species (Guo et al. 2022, 2024), and few have experimentally manipulated microbiome predator diversity to test sBEF relationships (Berlinches de Gea, Li, et al. 2023). Thus, despite their diversity and functional importance, the effects of increasing microbiome predator diversity on plant performance, underlying mechanisms, and potential interactions with N availability remain unclear.

We explore how increasing soil microbiome predator diversity interacts with varying N inputs to affect plant performance ( *Cannabis sativa* L.). To do so, we inoculated pots containing a sand‐potting soil mixture and *C. sativa* L. seedlings with four predator diversity levels (0 to 20 protist species +0 to 4 nematode species) across 4 N levels (0 to 150 kg N ha^−1^ year^−1^). To provide a broader picture of the sBEF relationship, we correlated several plant and soil parameters such as soil microbial biomass C and N, as well as total N in the soil, chlorophyll and plant nutrient content (here used to integrate the measurements of leaf C, leaf N, leaf S and leaf C/N ratio) and identified bacterial diversity by amplicon sequencing. Additionally, we used Structural Equation Modelling (SEM) analysis to identify potential underlying mechanisms linking microbiome predator diversity to plant performance. We hypothesize that (1) the addition of microbiome predators, irrespective of diversity, increases plant biomass and nutrient content, particularly under low nitrogen input; (2) increasing microbiome predator diversity further enhances plant performance; (3) changes in plant biomass due to predator addition/diversity can be explained by shifts in plant and soil nutrient parameters as well as by the bacterial community composition.

## 2 Materials and Methods

### 2.1 Experimental Set‐Up

The greenhouse pot experiment was carried out at Wageningen University & Research, the Netherlands. It included a fertilization treatment with four levels (0, 25, 100 and 150 N kg ha^−1^ year^−1^) crossed with four microbiome predator diversity levels (0, 6, 12, and 24 microbiome predator species). Each of the 16 treatment combinations was replicated 10 times, resulting in 160 1 L pots. The soil in the pots was composed of a 1:1 mixture of sieved (2 mm) river sand and low‐nutrient potting soil (bought from Lensli, Bleiswijk) already available at Wageningen University greenhouse and previously autoclaved (121°C–136°C, 4.5 h, 2.5 bar; Scholz, Germany). While N treatment addition was chosen based on agricultural practices of hemp cultivation ( *C. sativa* cv. “Ivory”), according to the manufacturer's formulation the potting soil contained a small background fertilizer charge, including PG‐mix 15‐10‐20, indicating that the control substrate was not completely nitrogen‐free before the start of the experiment. However, this substrate was classified by the manufacturer as low nutrient, and the final 1:1 dilution with river sand further reduced the nutrient concentration. We therefore interpret the 0 N treatment as a non‐amended low‐nutrient baseline, against which additional N fertilization treatments of 25, 100, and 150 kg N ha^−1^ were applied. Additionally, assuming that inorganic N is 1%–5% of total N, depending on the mineralization of the organic material, most of our baseline N was present in organic forms and not immediately plant‐available. Each of the 16 treatment combinations was replicated 10 times, resulting in 160 1 L pots. N for our treatments was then added to the pots using ammonium nitrate (NH_4_NO_3_) (VWR Chemicals, Pennsylvania) 8 days after inoculation with the microbiome predators (See Section 2.2 for details on microbiome predators' inoculation). As our system was open due to the pot‐based experiment, some N loss through leaching may have occurred, although we attempted to minimize this by avoiding excessive watering and spillage. Gaseous losses, likely mainly as N_2_O, were probably of minor importance, as only a small proportion of N is typically lost as N_2_O under anoxic conditions, which were unlikely to persist for extended periods in our substrate. In addition, part of the N may have become immobilized within microbial biomass and necromass. However, these processes were not quantified, and therefore a complete N mass balance could not be established. The diversity levels were created as follows: 0 species of protists and nematodes; 5 species of protists and 1 nematode species; 10 species of protists and 2 of nematodes; 20 species of protists and 4 of nematodes. Pots containing 0 species of microbiome predators were used as a control for the diversity levels and pots with 0 microbiome predators and 0 N kg ha^−1^ year^−1^ were used as control for all treatments (Figure S1).

### 2.2 Inoculation of Soil Biota

#### 2.2.1 Microbiome Treatment

Suspensions of a standardized, diverse community of bacterial and fungal cultures were added into plastic bags containing the soil used for the experiment (See Section 2.3.1 for suspension preparation details). These bacterial and fungal suspensions were divided over the bags containing soil proportional to the bag's weight, using an accu‐jet pro pipette (Brand, Wertheim). As there were 30 bags of soil varying between 7.5 and 12.5 kg, each bag was inoculated with 15–25 mL of bacterial suspension and 25–40 mL of fungal suspension accordingly. The same soil was used to fill up the germination trays (see Section 2.4 for germination conditions) and the pots that hosted the seedlings for the rest of the experiment.

#### 2.2.2 Microbiome Predator Treatment

Protists and nematodes (See Section 2.3.2 for microbiome predator isolation and cultivation) were added to the pots 48 h after the seedlings were transferred to the pots (see Section 2.4 for germination conditions). To maintain consistent abundance (approx. 1700 protist and 7000 nematodes per pot) across the three diversity treatments, both protists and nematodes were added in a 1:0.5:0.25 volume ratio among the treatments (5, 10, 20 protist species; 1, 2, 4 nematode species) using a regular pipette. With this setup, protist and nematode species richness varied, but abundances remained equal among treatments. Each microbiome predator community added was randomly assembled, ensuring that each replicate of the treatment had a unique set of microbiome predator taxa, thus avoiding keystone species effects (Banerjee et al. 2018). By creating one distinct microbiome predator community per replicate, we generated 40 different communities which were the same for the 4 N treatments. Consequently, each protist and nematode species was roughly equally distributed across all treatments, with each protist species present in 60–64 pots and 68–72 pots for nematodes. To account for the varying volumes of added suspensions, an equal amount of NMAS (the same buffer used to inoculate the microbiome predators) was added to the pots that did not receive any microbiome predators.

### 2.3 Preparation of Soil Biotic Treatments

#### 2.3.1 Microbiome Treatment

For bacteria, we used the same cultures created and used by Berlinches de Gea, Li, et al. (2023). Shortly, the cultures were extracted from seven different soils located in the surroundings of Wageningen (51°58′18″ N 5°41′02″ E; 51°58′44″ N 5°42′15″ E; 51°58′23″ N 5°43′05″ E; 51°58′57″ N 5°43′26″ E; 51°57′56″ N 5°38′33″ E; 51°57′16″ N 5°36′16″ E; 51°57′26″ N 5°36′08″ E) according to Geisen et al. (2022). Before inoculation, bacterial suspensions were adjusted to 0.1 optical density (OD600) using a Pharmacia Novaspec II spectrophotometer (Pharmacia Biotech Novaspec II, The Netherlands). Lastly, all subcultures were pooled into the same 500 mL Schott Duran glass bottle (DWK Life Sciences GmbH, Germany).

Fungal cultures used in this experiment were a subset of the cultures used by Berlinches de Gea, Li, et al. (2023) (*Acremonium* sp., *Alternaria alternata* , *Arthrinium phaeospermum*, *Chaetomium globosum*, *Fusarium oxysporum*, *Gibberella avenacea*, *Ilyonectria macrodidyma*, *Mucor hiemalis*, *Neonectria radicícola*, *Penicillium chrysogenum*, *Plectosphaerella cucumerina*, *Trichoderma citrinoviride*). While some of these cultures are facultative pathogens, the reason behind their selection was their well‐known trait as common inhabitants of soils and their previous use in other controlled experiments (Geisen et al. 2021). Additionally, we did not observe abundant disease symptoms in the cultured experimental plants. These cultures were kept in potato dextrose agar (PDA) containing 1 L demineralized water, 19 g of potato dextrose agar powder (OXOID, UK) and 7.5 g Select agar powder (Invitrogen, UK). We created a fungal suspension by scraping the entire surface off with 1 mL NMAS using a cell scraper (Greiner Bio‐One., The Netherlands). As for bacteria, all subcultures were pooled into a 1 L Schott Duran glass bottle (DWK Life Sciences GmbH, Germany).

#### 2.3.2 Microbiome Predator Treatment

Protist species used were a subset of the species cultures used by Berlinches de Gea, Li, et al. (2023) ( *Acanthamoeba castellanii* , *Acanthamoeba* sp., *Allovahlkampfia* group 1, *Allovahlkampfia* group 2, *Cercomonas lenta*, *Cercomonas* sp., *Cochliopodium minus*, *Didymium* sp., *Heterolobosea* sp. 1, *Heterolobosea* sp. 2, *Mycamoeba* sp., *Naegleria clarki*, *Naegleria* sp. 1, *Naegleria* sp. 2, *Rosculus terrestris*, *Spumella* sp., Unidentified ciliate, Unidentified cilliate 2, Unidentified cilliate 3, Unidentified flagellate, *Vahlkampfia bulbosis*, *Vannella* sp., *Vannella* sp. 2, *Vermamoeba vermiformis*). Shortly, protists were extracted from soils using a modified liquid aliquot method (LAM) as described by Geisen et al. (2014).

In addition, we used four species of bacterivorous nematodes (*Acrobeloides varius*, *Caenorhabditis remanei*, *Rhabditophanes* sp. and *Rhabditis* sp.) obtained from cultures available in the laboratory of Nematology, Wageningen University & Research. These cultures were grown in a Nematode Growth Medium containing 3 g NaCl, 2.5 g bacterial peptone, 17 g Agar, 975 mL water, 1 mL cholesterol, 1 mL 1 M CaCl_2_, 1 mL 1 M MgSO_4_, and 25 mL 1 M potassium phosphate and kept at 20°C. *Escherichia coli* (OP50) was added to each plate to feed the nematodes. To increase nematode abundance, 2–3 small pieces of the NGM cultures were transferred to a new NGM every 10 days. Before the inoculation, nematode cultures were washed with NB‐NMAS (Page 1976) liquid media, the same media used to maintain protist cultures.

### 2.4 Seed Germination

*Cannabis sativa* (variety “Ivory”) seeds were sown into a plastic‐covered tray containing autoclaved soil (121°C–136°C, 4.5 h, 2.5 bar; Scholz, Germany) inoculated with bacteria and fungi (See Section 2.1 for inoculation details). This soil did not contain microbiome predators. The plastic remained for 1 week to ensure the right germination conditions. After 2 weeks of growth, seedlings were planted in 1 L pots inoculated with the same bacterial and fungal community and composed of a 1:1 mixture of sieved (2 mm) and autoclaved (121°C–136°C, 4.5 h, 2.5 bar; Scholz, Germany) river sand and low‐nutrient potting soil (Lensli, Bleiswijk).

### 2.5 Greenhouse Conditions

Plants were grown for 42 days with a soil moisture content kept around 50% of the water‐holding capacity and air moisture content at 70%. The plants were exposed to a day/night regime of 16/8 h of artificial photosynthetically active radiation daylight (PAR, 400–700 nm) and 18°C/20°C. Plants were randomly rearranged every 2 weeks to avoid an effect caused by environmental heterogeneity in the greenhouse.

### 2.6 Harvest and Processing of Plant and Soil Samples

After 42 days of plant growth, plants and soil were harvested. Watering was stopped 4 days pre‐harvest to ease the process of soil removal from roots. Stems and leaves were cut off at the soil level using plant scissors. Plant biomass was measured after drying at 70°C for 120 h. The root system was separated from the soil by gently shaking while aiming to reduce root damage. Roots covered with soil particles were stored at 4°C, after which they were washed. Root biomass was then measured after drying for 48 h at 70°C. To determine soil moisture, 5–10 g of soil was collected, dried at 70°C for 144 h and re‐weighed. The relative leaf chlorophyll content was measured to analyze the plant performance using a SPAD portable chlorophyll meter (multispeQ, PhotosynQ) 1 day before harvest. Per plant, two fully developed leaves were measured. The leaves were chosen by counting four stories down from the youngest leaves on top. This way it could be ensured to measure leaves mature enough on each plant for an accurate representation of the chlorophyll content and young enough to not have fallen off or turned yellow with a loss of chlorophyll. On each leaf, the middlemost leaf of the palmate leaf was measured on the widest region. For pH measurements, 5–10 g of soil was suspended in a 2:5 ratio with demineralized water in a 50 mL falcon tube (Sarstedt; Numbrecht, Germany). The falcon tube was shaken for 2 h at 250 rpm (Multifuge 3 S‐R Refrigerated Centrifuge; Heraeus, US), after which pH was determined directly from the liquid using a soil pH meter with a glass electrode (SI analytics, Mainz). Soil samples were stored at 4°C for posterior nematode extraction and at −20°C for DNA extraction (see Section 2.7). An extra 100 g of soil per sample was stored at 4°C to conduct analyzes for microbial biomass carbon (MBC), microbial biomass nitrogen (MBN) and available NH_4_^+^ NO_3_^−^ content (see Section 2.8).

### 2.7 Nematode Counting, Soil DNA Extraction, Sequencing and qPCR

Soil nematodes were extracted using Oostenbrink elutriation (Oostenbrink 1960), stored in a glass 100 mL jar at 4°C and counted in a petri dish with gridlines (50 mm × 15 mm) under an IXplore Standard microscope at 100× magnification (Zeiss, Stuttgart) to determine potential cross‐contaminations. In fact, we found individuals of the nematodes used in the experiment in pots that were not inoculated with microbiome predators, indicating cross‐contaminations between pots and hindering the real potential implications of our results. Nevertheless, the significant differences found among treatment levels suggest that the initially inoculated microbiome predator communities were efficiently established and impacted plant performance throughout the experiment, aligning with the priority effect theory (Debray et al. 2021).

DNA was isolated from 1 g of soil as described by Wilschut et al. (2019) with the only difference of using 1.5 g of silicon carbide powder (ThermoFisher, Kandel, Germany) instead of 3 mL of bead solution and iron beads. The extracted DNA samples were stored at −20°C. DNA concentration was then quantified with a Qubit Fluorometer device (Thermo Fisher, USA) by using a dsDNA Broad Range kit (Thermo Fisher, USA) and sent to Genome Quebec (Quebec, Canada) for 2 × 300bp pair‐end NextSeq sequencing, using the primers 515 F and 806 R, which target the V4 region of the 16S rRNA gene (Wu et al. 2015). Samples obtained from DNA extraction were also amplified and quantified using qPCR (CFX Opus 96; Thermo Fisher, USA) for the bacterial and fungal DNA presence, as well as their ratio (B: F). Per 3 μL sample template, 1 μL of forward and reverse primer were added, along with 5 μL of sterilized Milli‐Q water and 10 μL of iQTM SYBR Green Supermix (Bio‐Rad, Hercules, USA). For bacteria, the primer pair was 515 F and 806 R, which target the V4 region of the 16S rRNA gene (Wu et al. 2015), while for fungi we used the primer combination FF390.1 and FR1 targeting the V7‐V8 region of the 18S rRNA gene (Verbruggen et al. 2012). The PCR conditions were as follows: Denaturation at 94°C for 3 min, followed by 35 cycles of 95°C for 15 s, 56°C for 10 s, and an extension step at 72°C for 30 s. Then, the bacterial and fungal DNA proportions in the soil were calculated from a standard linear regression curve obtained for each primer pair by making a dilution series of a solution containing a known concentration of DNA isolated from the soil and measuring the respective cycle threshold (Ct) values. For bacteria, this formula was *Y* = −3.62*x* + 16.58. For fungi, this formula was *Y* = −3.90*x* + 18.95. The Ct cut‐off was set at 50 Relative Fluorescence Units (RFU) to filter out signals that were too weak and to identify signals that came up too late (Ct > 30).

### 2.8 Microbial Biomass and Soil Nutrient Content Determination

Microbial biomass C (MBC) and N (MBN) were determined using fumigation‐extraction (Brookes et al. 1985; Vance et al. 1987). Fumigation was carried out in desiccators for 24 h at 25°C. Fumigated and non‐fumigated samples of 10 g moist soil were extracted with 0.5 M K_2_SO_4_ at 200 rpm for 30 min. A multi‐N/C 2100S analyzer was used to measure organic carbon (OC) and total nitrogen contents in the extracts. Microbial biomass C was calculated using the formula: (Extracted organic C from fumigated soil − Extracted organic C from non‐fumigated soil)/0.45 (correction factor) for MBC. MBN was calculated similarly: (Extracted organic N from fumigated soils − Extracted N from non‐fumigated soil)/0.54 (correction factor) for MBN.

### 2.9 Statistical Analysis

#### 2.9.1 Plant Parameters

All statistical analyzes were performed in R software ver. 4.2.2 (Team 2013). Figures were created using the package ggplot2 (Wilkinson 2011) and finalized using Adobe Illustrator ver. 27.9.4 (https://www.adobe.com/products/illustrator.html). Before the analyzes of treatment effects, we checked for the residual normality and homoscedasticity of the models by visually inspecting the model assumptions using the “plot” function. To test for the effect of predator addition on plant performance and soil biochemistry, we constructed mixed linear models for each plant and soil parameter measured using the “nlme” package (Pinheiro et al. 2022), with “predator addition,” “nitrogen addition” and their interactions as fixed factors, and the “microbiome predator community” and “microbiome predator diversity” as random intercepts. The term “microbiome predator community” refers to the 40 predator communities added to the experiment and “microbiome predator diversity” refers to the diversity levels used. We then examined the significance using type II Anova for the different models created for each plant and soil parameter.

To test the effect of both an increasing diversity of predators and an increasing N inputs as well as their interaction on plant performance, we modelled both treatments as numerical variables. We again created a mixed linear model for each plant and soil parameter measured using the “nlme” package (Pinheiro et al. 2022) and adding “microbiome predator community” as a random factor. To account for increases in response variance to increasing levels of microbiome predator diversity and nitrogen inputs, we allowed the model to estimate variance associated with our explanatory variables “microbiome predator diversity” and “nitrogen input” using the “varExp” function.

#### 2.9.2 Bacterial Community Analyzes

The raw data obtained from the bacterial sequencing was processed using QIIME2 (Bolyen et al. 2019) to remove chimeras, quality filtering, and finally obtain an ASV table. We used the SILVA database for taxonomic annotation of ASVs. The removal of primers and demultiplexing was performed by Genome Quebec (Quebec, Canada). We managed significant variations in read counts by excluding samples that deviated from the average read count by more than ±2 standard deviations, which led to the removal of six samples with less than 12,122 reads and one sample with more than 37,433 reads. Following this, we eliminated all ASVs that appeared in fewer than four samples and had a maximum relative abundance below 0.01% to minimize the impact of rare ASVs on the community composition.

Following Deng et al. (2024) we only measured Shannon diversity as a proxy for alpha diversity for which we used the “alphaDiversity” function from the “vegan” package (Oksanen et al. 2013). NMDSs were performed using the function “metaMDS” from the “vegan” package (Oksanen et al. 2013), using the following parameters: *k* = 3, distance = “bray”, trace = FALSE, autotransform = FALSE.

#### 2.9.3 Functional Annotation

To detect how changes in bacterial community linked to changes in the functionality of the system, a functional annotation of the bacterial community was performed by using the “microeco” package (Liu et al. 2020) and using the FAPROTAX database (Louca et al. 2016). These functions are assigned based on literature descriptions and cultured representatives and have been widely used to infer potential bacterial functions from 16S rRNA gene data in soil studies (Clarke et al. 2026; Zhang et al. 2026). Because FAPROTAX predictions are based on taxonomy rather than direct measurements of activity, the inferred functions should be interpreted as indicators of potential functional capacity rather than evidence that these processes were actively occurring in the studied communities.

The bacterial community was first rarefied using the lowest sum of reads per sample, which was 13,695. This process resulted in the removal of 3 ASVs. Then, the functions were filtered in two ways: First, the ones with less than 0.1% of relative abundance were removed from the database. Secondly, we only selected the functions that might be linked to our experimental setup, ending up with the following functions: “Aerobic chemoheterotrophy,” “anaerobic chemoheterotrophy,” “cellulolysis,” “dark hydrogen oxidation,” “dark oxidation of sulfur compounds,” “fermentation,” “hydrocarbon degradation,” “intracellular parasites,” “nitrogen respiration,” “predatory or exoparasitic,” “respiration of sulfur compounds,” “ureolysis.”

Once function annotation was done and filtered, normality was assessed using the Shapiro–Wilk test. If data met the assumptions of normality and homogeneity of variances, parametric tests were used: Student's *t*‐test for two‐level comparisons (*PredatorsAdded*) and ANOVA for multi‐level factors (*NitrogenLevels* and *SpeciesNumber*). If assumptions were violated, non‐parametric alternatives were applied: Wilcoxon rank‐sum test or Kruskal‐Wallis test, respectively.

Additionally, effect sizes were estimated by extracting *R* ^2^ values from ANOVA models as a measure of explanatory power or, in cases where normality was not met, a pseudo‐*R* ^2^ (ℇ^2^) was estimated as analogous to *R* ^2^. As FAPROTAX predicts potential functions based on assigned taxonomy, these results represent inferred rather than directly measured functions.

#### 2.9.4 Differential Abundance Analyzes

In order to identify some potential genus of bacteria that might be influencing the response of the plant biomass, we performed differential abundance analyzes using DESeq2 package in R (Love et al. 2014). Additionally, we only showed the genera that were statistically significant (*p* < 0.05) and visually highlighted only genera with a log2 median ratio bigger than ±2. The differential abundance analyzes comparisons were done among predator addition (Yes vs. No) and predator diversity (all the pair combinations between treatments of 0, 6, 12 and 24 species). The results of those analyzes are depicted in the figures (*p* > 0.05 = ns; *p* < 0.05 = *; *p* < 0.01 = **; *p* < 0.001 = ***).

#### 2.9.5 Correlation Analyzes

To obtain correlations between all the variables and parameters measured we used Pearson correlations with the function “rcorr” from the “Hmisc” package (Harrell Jr and Harrell Jr 2019). Prior to the correlations, binary categorical variables (e.g., “Yes”/“No”) were converted to numeric (0/1) format for inclusion in the correlation matrix. To correct for multiple testing across all pairwise correlations, False Discovery Rate (FDR) correction was applied using the Benjamini‐Hochberg procedure. A corrected *p*‐value threshold of 0.05 was used to assess significance. For the correlation matrix plot, we used the “corrplot” package (Wei et al. 2017).

#### 2.9.6 Structural Equation Modelling (SEM)

To further examine how independent and interactive impacts of nitrogen addition and microbiome predator diversity directly or indirectly, through changes in microbial communities, ultimately affected plant biomass, we constructed a structural equation model using the piecewise SEM package (Lefcheck 2016). We hypothesized that N inputs, microbiome predator diversity, and their interaction could directly affect microbial N (mg/kg) and bacterial community composition (first NMDS axis). Within the soil microbial community, we hypothesized that bacterial community composition affected microbial N (mg/kg). Finally, we hypothesized that total plant biomass may be driven by bacterial community composition, changes in microbial N, as well as by the direct effect of nitrogen addition. In this model, we natural‐log‐transformed total plant biomass, microbial N (mg/kg), nitrogen addition (Ln + 1), and microbiome predator diversity (Ln + 1). Microbiome predator community was removed as a random effect after its initial addition, as it did not explain variation.

## 3 Results

### 3.1 The Addition of Microbiome Predators Affects Plant Growth but Not Nutrient Content

**FIGURE 1:**
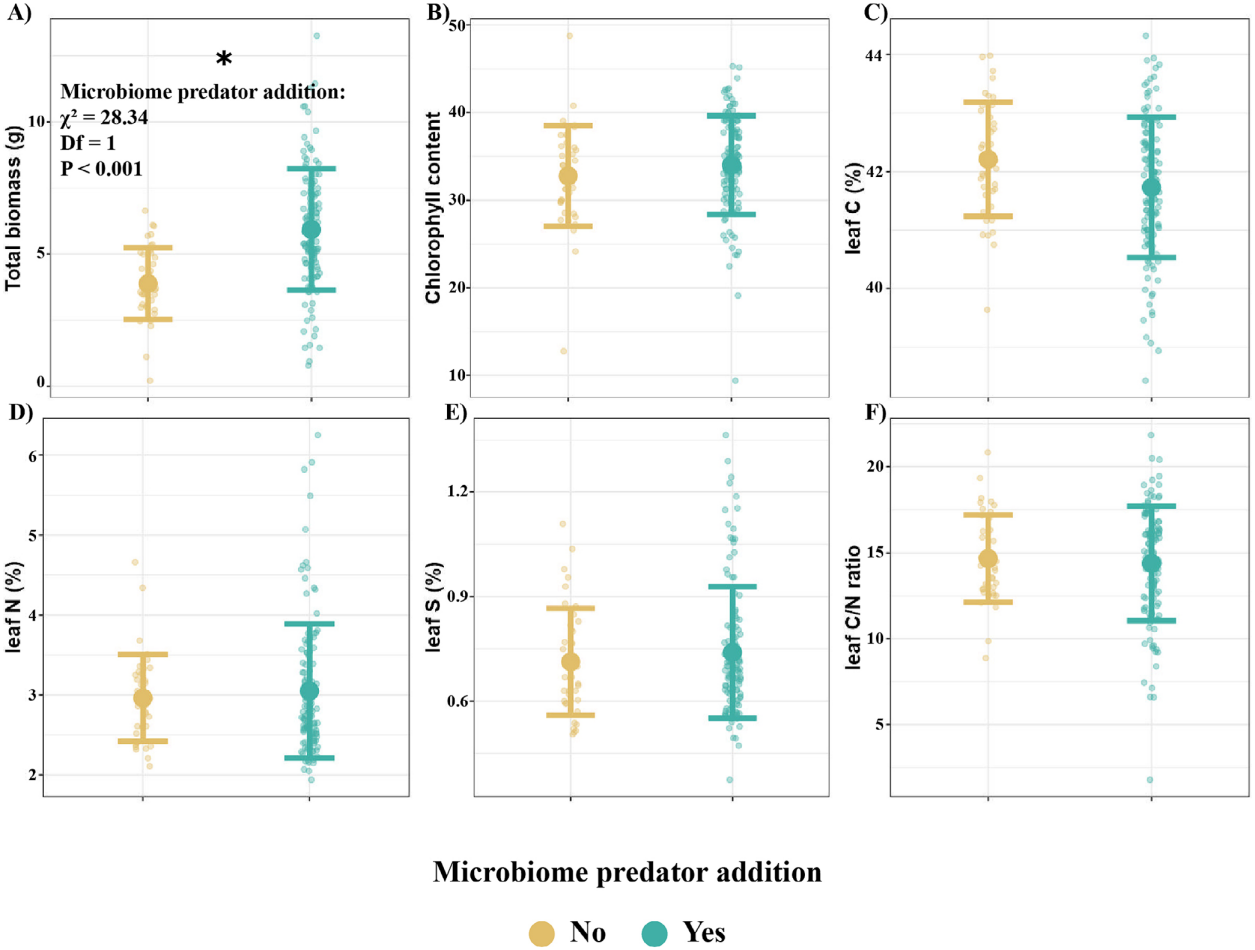
Microbiome predator addition increases total plant biomass (g) (A) but not chlorophyll content (B), leaf C (C), leaf N (D), leaf S (E), leaf C/N ratio (F) irrespective of diversity levels (X‐axis shared for all panels). Small dots represent the raw data, error bars represent the standard deviation, and the thicker dot represents the mean. Statistically significant effects (ANOVA; *p* < 0.05) are shown with an asterisk.

Overall, microbiome predator addition, independent of diversity levels, increased total plant biomass by 53% (*χ* ^2^ = 28.34; df = 1; *p* < 0.001; Figure 1A). This positive effect on plant growth was shown for both shoot (52%, *χ* ^2^ = 13.74; df = 1; *p* < 0.001; Figure S2A) and root biomass (66%, *χ* ^2^ = 21.2; df = 1; *p* < 0.001; Figure S2B). The root‐shoot ratio was not affected by any of the treatments (*χ* ^2^ = 0.45; df = 1; *p*‐value = 0.5; Figure S2C). In contrast to plant biomass, microbiome predator addition did not affect chlorophyll and plant nutrient content parameters (leaf C, leaf N, leaf S and leaf C/N ratio) (*p* > 0.05; Figure 1).

**FIGURE 2:**
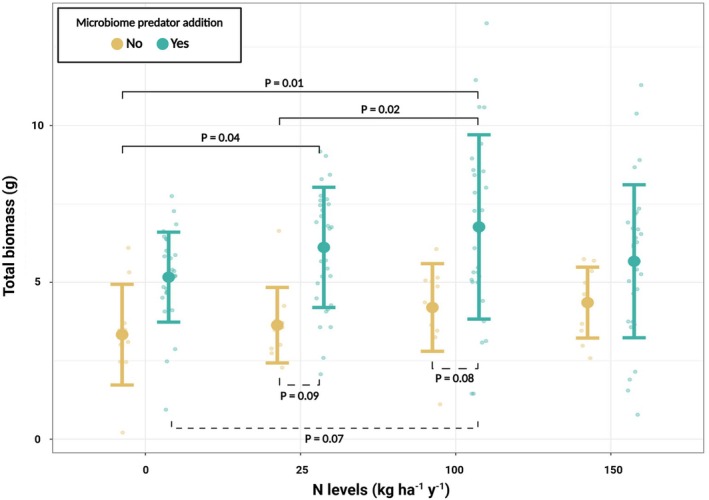
Microbiome predator addition, irrespective of diversity levels, enhances plant total biomass (g) (Y‐axis), reaching its maximum at low and mid N levels (X‐axis). Small dots represent the raw data, error bars represent the standard deviation, and thicker dots represent the mean. Lines connecting treatments represent significant (continuous line) or marginally significant (0.05–0.1; dashed line) post hoc Tukey tests.

The positive effect of microbiome predator addition, irrespective of diversity levels, on plant biomass was not related to the amount of N added (*p* = 0.61; *χ* ^2^ = 1.81, df = 1; Figure 2). However, the amount of fungal DNA and bacteria to fungi ratio did respond to an interaction between microbiome predator addition and the amount of N added (*p* < 0.001; *χ* ^2^ = 40.505, df = 1; *p* < 0.001, *χ* ^2^ = 8.84, df = 1 respectively; Figure S3) in which the effect of microbiome predator addition was enhanced at higher levels of N inputs.

### 3.2 Total Plant Biomass Depends on Both Increasing Diversity of Microbiome Predators and Nitrogen Input Levels

**FIGURE 3:**
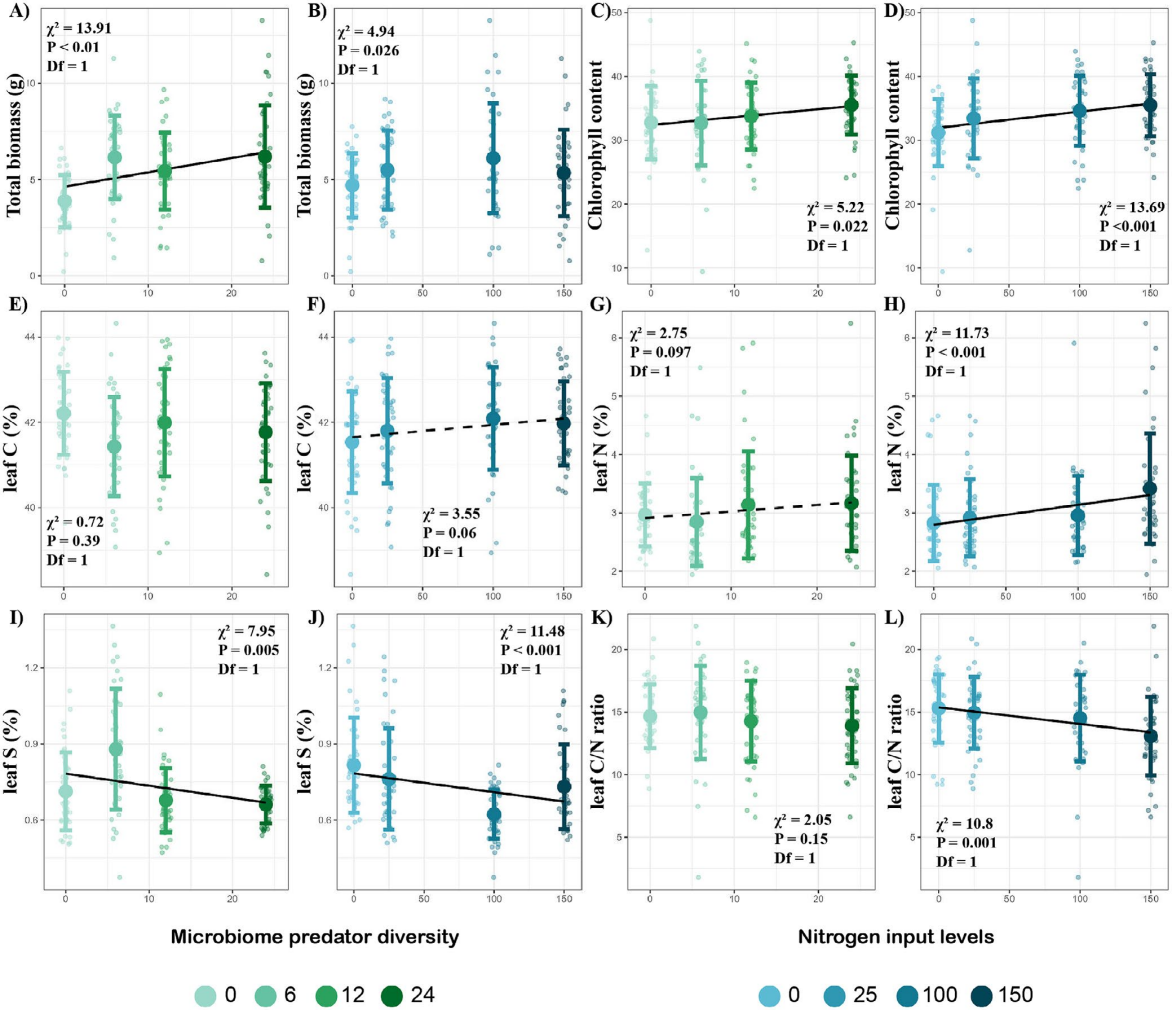
The relationship of plant total biomass (g) (A, B), Chlorophyll content (C, D), leaf C (%) (E, F), leaf N (%) (G, H), leaf S (%) (I, J), and leaf C/N ratio (K, L) with an increasing diversity of microbiome predators (A, C, E, G, I, K) or an increasing N input (B, D, F, H, J, L). Small dots represent the raw data; error bars represent the standard deviation, and the thicker dot represents the mean. The test results shown in the panels are based on the linear mixed‐effect models performed for each plant parameter. The X‐axis is shared per column.

To understand the relationship between microbiome predator diversity and plant performance, we analyzed the impact of increasing diversity of microbiome predators (0–20 protist species; 0–4 nematode species) on different plant parameters related to plant biomass and chlorophyll and leaf nutrient content. We found that increasing microbiome predator diversity significantly increased plant biomass up to 60% (Figure 3; *χ* ^2^ = 13.91, df = 1, *p* < 0.001). Increasing nitrogen addition had a weaker positive effect on plant biomass (up to 30%, *χ* ^2^ = 4.94, df = 1, *p* = 0.026). For chlorophyll content, both predator diversity (*χ* ^2^ = 5.22, df = 1, *p* = 0.022) and N addition (*χ* ^2^ = 13.69, df = 1, *p* < 0.001) increased its content by 8% and 14%, respectively. No significant interaction between predator diversity and N addition was detected (ANOVA; all *p* > 0.05). Leaf C (%) (Figure 3E), leaf N (%) (Figure 3G), and leaf C/N ratio (Figure 3K) were not significantly (*p* > 0.05) affected by increasing microbiome predator diversity. Except for total plant biomass, increasing nitrogen levels (as shown by *p* and *χ* ^2^) affected plant parameters more strongly than microbiome predator diversity.

While no interactive effect of microbiome predator diversity and N input levels on plant biomass was found, they interactively affected plant biomass when N input levels were modelled as a factor (Wald χ^2^ test; *p* < 0.001; *χ* ^2^ = 18.07; Figure S4).

### 3.3 Nitrogen Input Levels Rather Than Microbiome Predator Diversity Influence Soil Biochemical Properties

**FIGURE 4:**
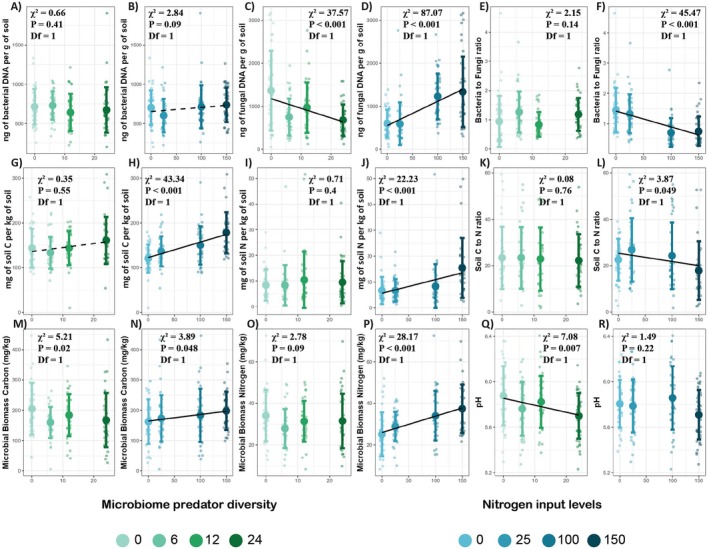
The relationship of bacterial (A, B) and fungal (C, D) DNA in soil, bacteria to fungi ratio (E, F), soil C (G, H), soil N (I, J), soil C to N ratio (K, L), microbial biomass C (M, N) and N (O, P) and pH (Q, R) with an increasing diversity of microbiome predators (A, C, E, G, I, K) or an increasing N input (B, D, F, H, J, L). Small dots represent the raw data, error bars represent the standard deviation, and the thicker dot represents the mean. The test results shown in the panels are based on the linear mixed‐effect models performed for each plant parameter. The X‐axis is shared per column.

To assess how microbiome predator diversity and nitrogen input influenced soil biochemical properties, we tested their effects on parameters related to bacterial and fungal abundance as well as on soil nutrient content (Figure 4). Increasing microbiome predator diversity significantly reduced fungal abundance (*χ* ^2^ = 37.57, *p* < 0.001), microbial biomass carbon (MBC; *χ* ^2^ = 5.21, *p* = 0.02), and soil pH (*χ* ^2^ = 7.08, *p* = 0.007), corresponding to decreases of approximately 50%, 18%, and 3%, respectively, between the lowest and highest diversity levels.

Increasing N input levels strongly increased fungal abundance (*χ* ^2^ = 87.07, *p* < 0.001), total soil C and N (*χ* ^2^ = 43.34 and 22.23, respectively; both *p* < 0.001), and microbial biomass nitrogen (MBN; *χ* ^2^ = 28.17, *p* < 0.001) by 122%, 48%, 130%, and by 49% respectively, between the lowest and highest diversity levels. On the contrary, increasing N input levels reduced the bacteria‐to‐fungi ratio (*χ* ^2^ = 45.47, *p* < 0.001) and soil C:N ratio (*χ* ^2^ = 3.87, *p* = 0.049) by 48% and by 20% respectively, between the lowest and highest diversity levels.

### 3.4 Stronger Microbiome Predator Than N Input Effects on Bacterial Community Composition

**FIGURE 5:**
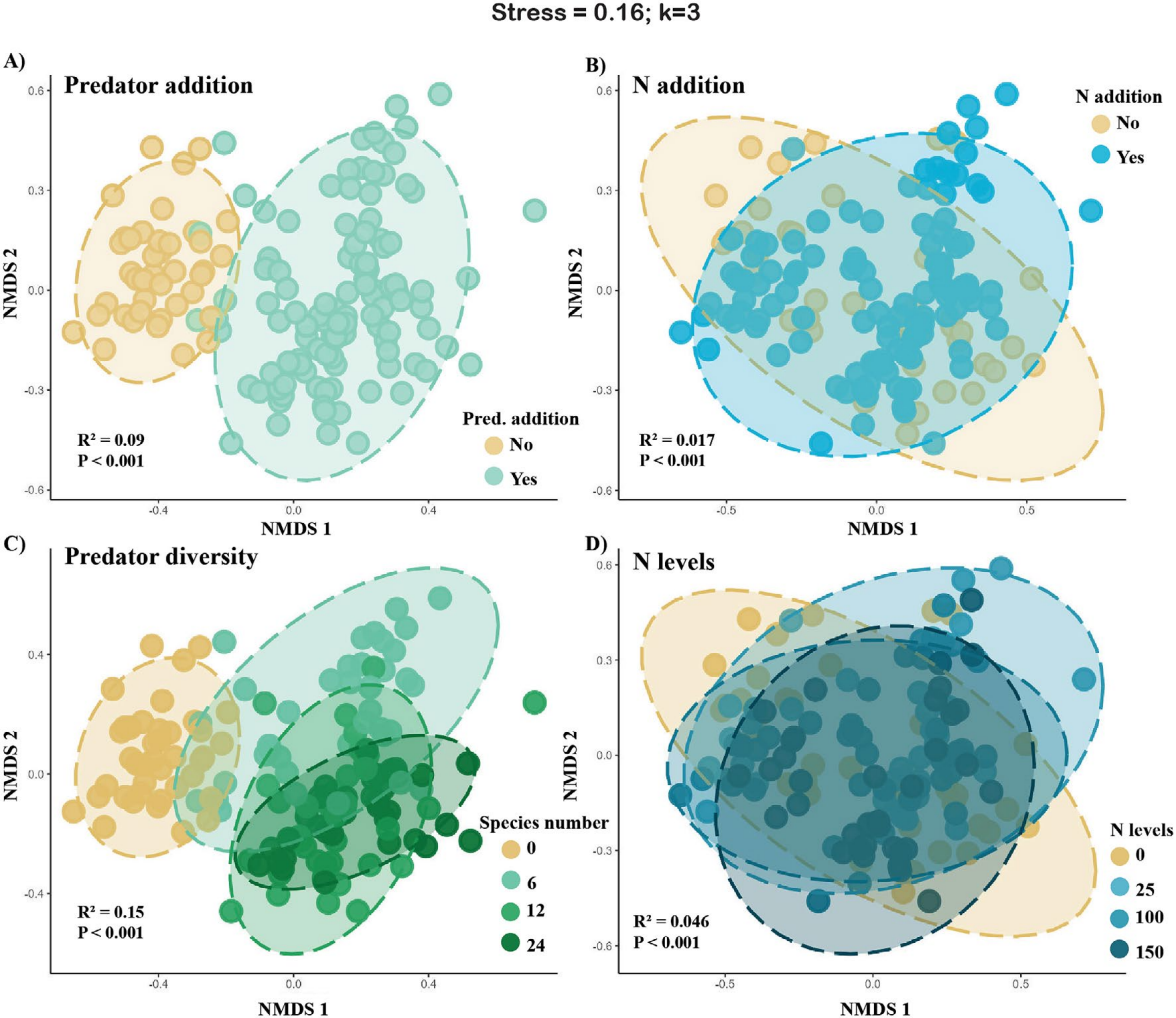
NMDSs representing the bacterial community composition at the ASV level depending on predators (A) or N (B) addition, as well as on an increasing diversity of predators (C), or an increasing level of N added (D), showing that microbiome predators had stronger effects on bacterial community composition than nitrogen inputs. The test results shown in each of the panels refer to PERMANOVA analyses. Stress and *k* values, indicating the overall fit of the data and the number of NMDS axes used within the ordination space, respectively, are indicated.

We analyzed the bacterial community composition as one of the main potential mechanisms underlying the impacts of predator and N addition, as well as of microbiome predator diversity and N input levels, on plant performance. The bacterial community composition was mostly affected by the sole addition of microbiome predators as well as by their diversity (PERMANOVA; *p* < 0.001; *F*‐value = 15.5; *R* ^2^ = 0.09; Figure 5A, *p* < 0.001; *F*‐value = 8.99; *R* ^2^ = 0.15; Figure 5C respectively) but also in a lower manner by N addition and N input levels (PERMANOVA; *p* < 0.001; *F*‐value = 2.4; *R* ^2^ = 0.017; Figure 5B, *p* < 0.001; *F*‐value = 2.39; *R* ^2^ = 0.046; Figure 5D respectively).

While bacterial community composition was mainly shaped by microbiome predator addition, alpha diversity (Shannon index) did not change with the addition of microbiome predators or N (*p* > 0.05 Figure S5), but it did change with the interaction between microbiome predator diversity and N input levels, with an increasing diversity reducing alpha diversity at higher N input levels (ANOVA; *χ* ^2^ = 8.11; df = 1; *p* < 0.01; Figure S6).

### 3.5 Differential Abundance Analyzes

To detect bacterial groups that were most strongly linked with changes in plant performance, we performed differential abundance analyzes. Microbiome predator addition increased the abundance (log2 fold change > 2) of 19 genera, while it decreased the abundance (log2 fold change < −2) of 4 genera (Figure S7). The three genera that increased the most were *Alicyclobacillus* (6.76 log2 fold change), *Filibacter* (5.61 log2 fold change), and *Rugamonas* (5.57 log2 fold change). The ones that decreased the most were *Daeguia* (−2.17 log2 fold change), *Leptospira* (−2.54 log2 fold change), and *Paludibaculum* (−3.14 log2 fold change).

When comparing treatments with different levels of predator diversity, the largest number of genera showing log2 fold changes > 2 or < −2 occurred in the comparisons between the predator addition treatments (6, 12, or 24 species) and the control treatment with no predators (0 species), while also several taxa were differentially abundant between the diversity levels (Figure S8).

### 3.6 Functional Annotation of Bacterial Communities

**FIGURE 6:**
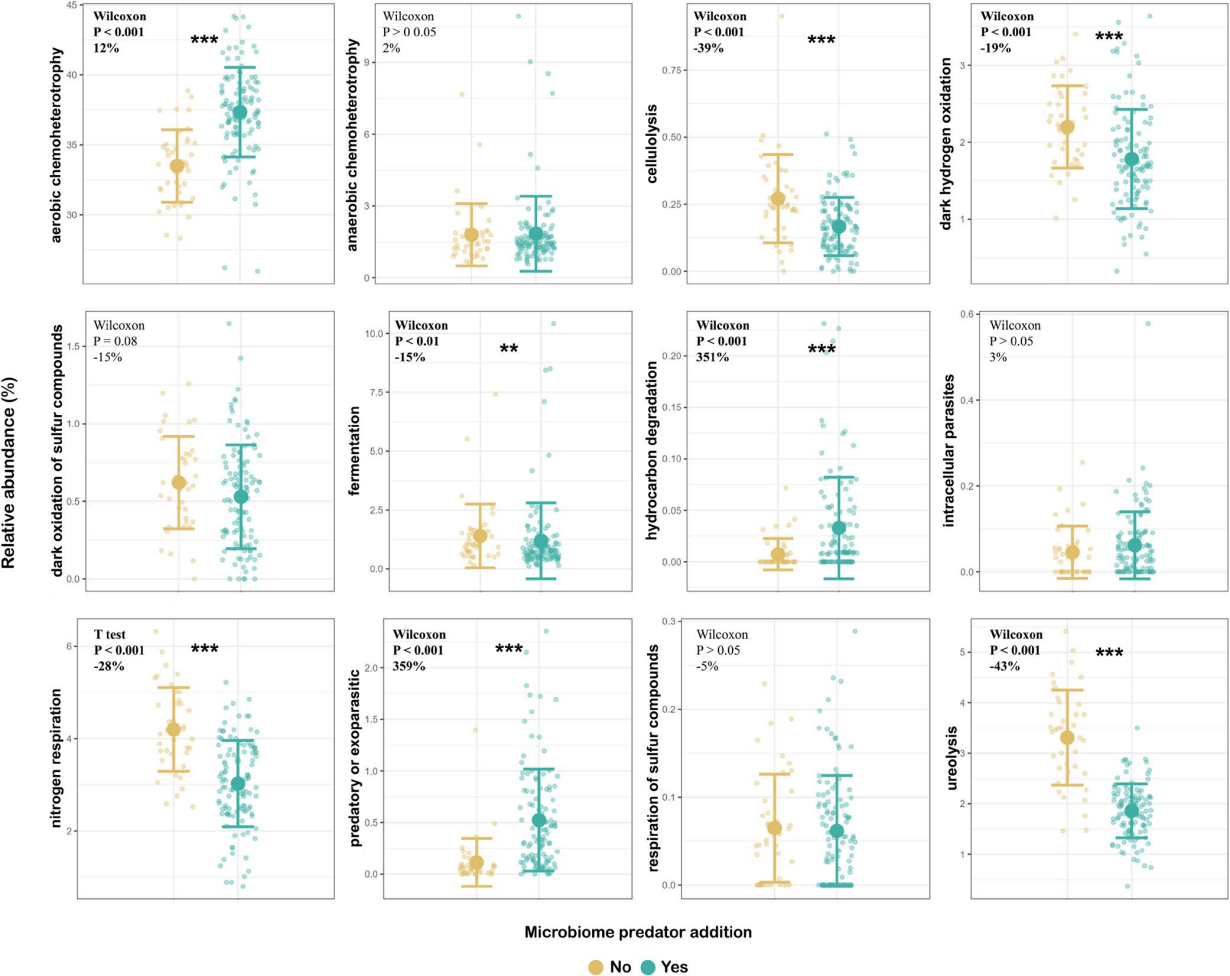
Boxplots depicting the effect of predator addition on the relative abundance of the different bacterial functions. Statistical tests performed, together with the *p* value, the *R* ^2^ or a pseudo‐*R* ^2^ (ℇ) and the percentage changes are shown within each panel. Percentage changes are calculated as the average differences between the 0 and 24 species treatments.

To determine whether the changes in bacterial composition were linked to potential changes in bacterial‐driven functions, we functionally annotated the bacterial taxa (using FAPROTAX (Louca et al. 2016)) and focused on 12 functions related to plant performance as well as N and C cycling. Among those 12, microbiome predator addition significantly affected 8 of them (Figure 6), as it increased “aerobic chemoheterotrophy” (11.51%, Wilcoxon; *p* < 0.001), “predatory or exoparasitic” (359%, Wilcoxon; *p* < 0.001) and “hydrocarbon degradation” (350.6%; Wilcoxon; *p* < 0.001), while reducing “cellulolysis” (−38.5%, Wilcoxon; *p* < 0.001) “dark oxidation of sulfur compounds” (−18.9%, Wilcoxon; *p* < 0.001), “fermentation” (−15%; Wilcoxon; *p* < 0.01), “nitrogen respiration” (−28%, *T*‐test; *p* < 0.001) and “ureolysis” (−43%, Wilcoxon; *p* < 0.001).

**FIGURE 7:**
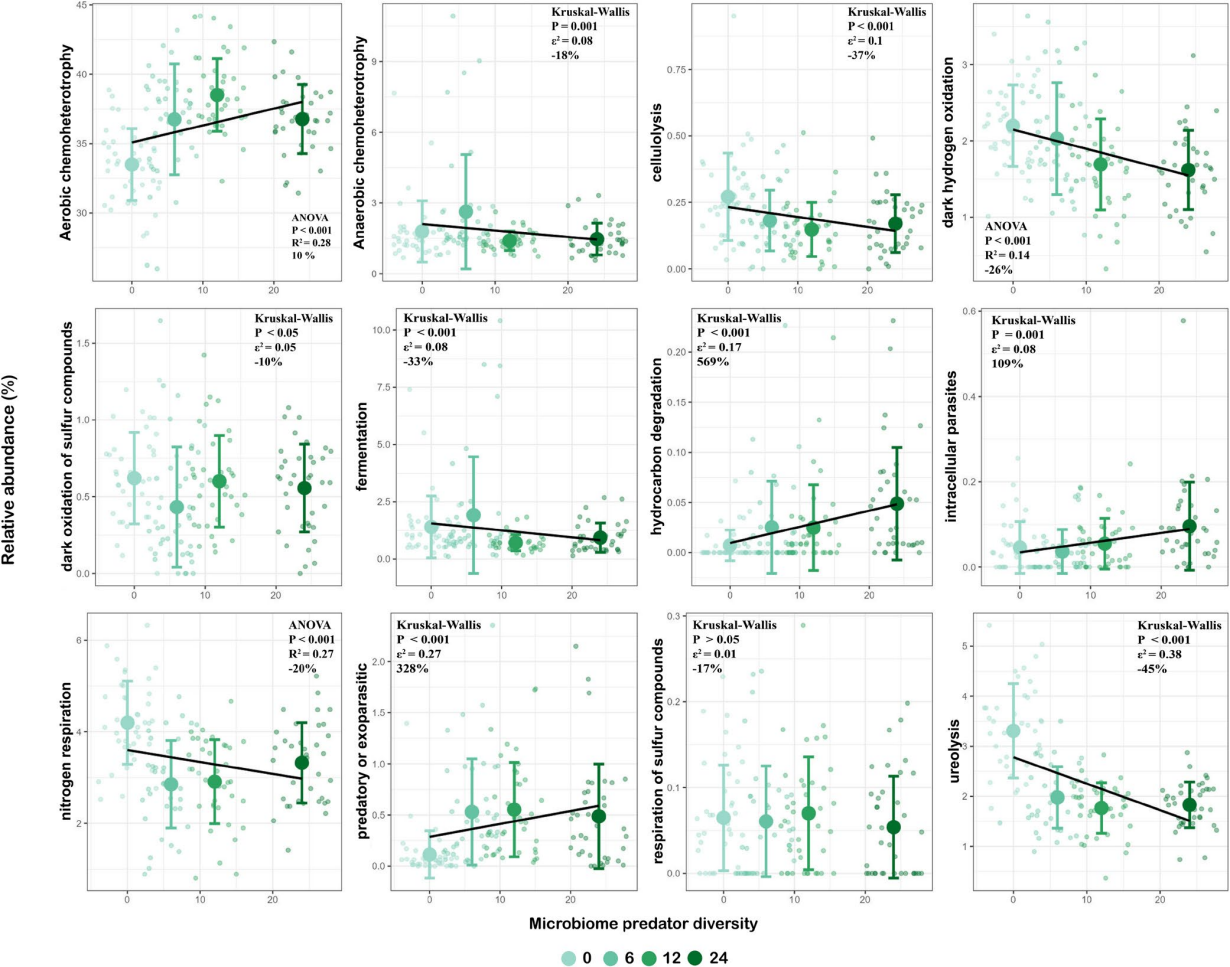
Boxplots depicting the effect of increasing protists diversity on the relative abundance of the different bacterial functions. Linear trends are shown only when the statistical tests were significant (*p* < 0.05). Statistical tests performed, together with the *p* value, the *R* ^2^ or a pseudo‐*R* ^2^ (ℇ) and the percentage changes are shown within each panel. Percentage changes are calculated as the average differences between the 0 and 24 species treatments.

When testing the effect of microbiome predator diversity on the same 12 functions, all but the respiration of sulfur compounds were affected (Figure 7). “Aerobic chemoheterotrophy” (*R* ^2^ = 0.28; ANOVA; *p* < 0.001), “hydrocarbon degradation” (ℇ^2^ = 0.17, Kruskal‐Wallis; *p* < 0.001), “intracellular parasites” (ℇ^2^ = 0.08, Kruskal‐Wallis; *p* = 0.001) and “predatory or exoparasitic” (ℇ^2^ = 0.27, Kruskal‐Wallis; *p* < 0.001) functions were enhanced compared to control, while “cellulolysis” (ℇ^2^ = 0.1, Kruskal‐Wallis; *p* < 0.001), “dark hydrogen oxidation” (*R* ^2^ = 0.14; ANOVA; *p* < 0.001), “fermentation” (ℇ^2^ = 0.08, Kruskal‐Wallis; *p* < 0.001), “nitrogen respiration” (*R* ^2^ = 0.27; ANOVA; *p* < 0.001) and “ureolysis” (ℇ^2^ = 0.38, Kruskal‐Wallis; *p* < 0.001) decreased. Importantly, the most abundant functions in the communities were “aerobic chemoheterotrophy” and “ureolysis,” which showed an increase of approximately 10% and a decrease of 45%, respectively, from the lowest diversity treatment to the highest.

### 3.7 Structural Equation Model (SEM)

**FIGURE 8:**
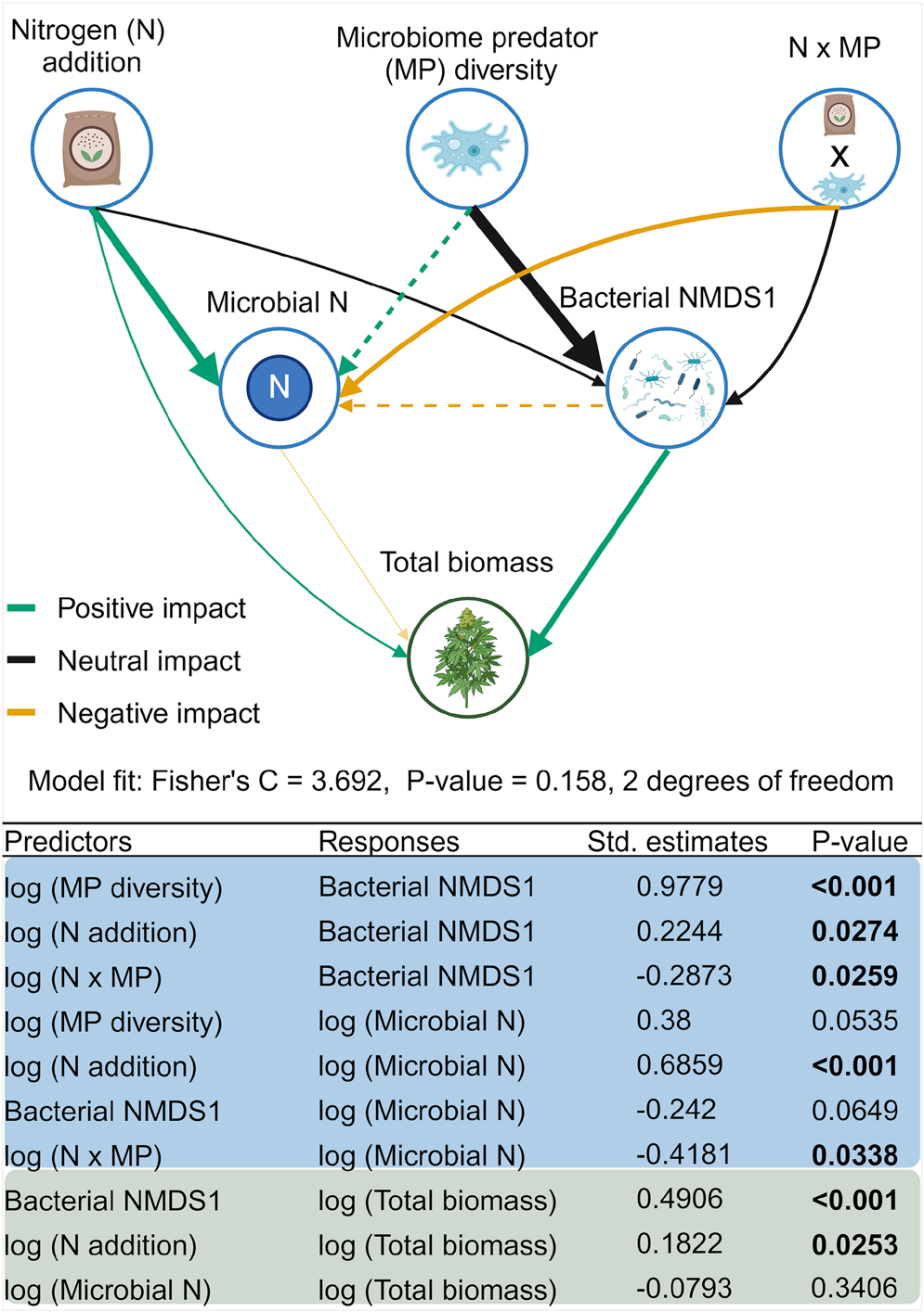
In the upper part, the SEM model is visualized, in which it is observed that plant biomass is most affected by bacterial community composition, which is most affected by microbiome predator diversity. Arrow widths are determined by their standardized estimates, while arrow colors depict the type of relationship (positive, negative or neutral). “Neutral” denotes types of relationships with non‐definable directional changes. Dashed lines represent marginally significant paths (0.05 < *p* value < 0.07). The bottom part shows the statistics of the model for each path. The colors of the table are linked to the colors on the circles surrounding each icon in the scheme above.

To statistically test the potential mechanisms through which microbiome predator diversity and N input levels affected plant biomass, we constructed an SEM (Figure 8). As we acknowledge the hypothetical effect of some other variables not used in the SEM, we performed a correlation analysis with all the variables measured in the study to visualize an overall picture (Figure S9). Our structural equation model presented a good fit (*p* = 0.158; Fisher's C = 3.692; 2 d.f), showing that N input levels and microbiome predator diversity, solely and interactively, affected total plant biomass through impacts on microbial N and bacterial community composition. Both N input levels (Std. estimate = 0.68; *p* < 0.001) and microbiome predator diversity (Std. estimate = 0.38; *p* = 0.054) separately had a positive effect on MBN (Microbial Biomass Nitrogen), but the interaction of both negatively affected MBN (Std. estimate = −0.41; *p* = 0.03). These changes in MBN were not directly associated with variation in plant biomass within the SEM framework, but N input levels directly increased plant biomass (Std. estimate = 0.18; *p* = 0.02). N input levels, microbiome predator diversity and the combination of both treatments significantly affected the bacterial community composition. Among these three, microbiome predator diversity was the main factor determining bacterial community composition (Std. estimate = 0.98; *p* < 0.001), which was the main variable affecting plant biomass (Std. estimate = 0.49; *p* < 0.001) (Figure 8).

## 4 Discussion

To our knowledge, this is the first study experimentally manipulating both protist and nematode diversity across multiple nitrogen regimes to test soil biodiversity–ecosystem functioning relationships in relation to plant performance. We here demonstrate that increasing soil microbiome predator diversity can enhance plant biomass without affecting chlorophyll and plant nutrient content, while nitrogen input levels predominantly improve chlorophyll as well as soil biochemical and plant nutrient contents. Overall, microbiome predator addition increased plant biomass by 53%, with the effect depending on the diversity level of predators. More specifically, we found that an increasing microbiome predator diversity enhanced plant growth up to 60% by shaping bacterial community composition and, consequently, potential ecosystem functions related to N cycling.

### 4.1 Microbiome Predator Addition on Plant Performance

Microbiome predator addition, irrespective of diversity levels, increased plant biomass by about half without affecting chlorophyll and plant nutrient content. These findings are partially in line with our first hypothesis and previous studies based on bacterivorous protists or nematodes (Krome et al. 2009; Saleem et al. 2012; Wilschut and Geisen 2021). Although the positive effect of microbiome predator addition on plant biomass was observed across all nitrogen (N) levels and there was no interaction between microbiome predator addition and N inputs, the effect appeared more pronounced under low and medium N addition (Figure 2). This trend may suggest that N limitation was alleviated in the highest N treatments, reducing the relative benefit of predator addition. However, given the marginal statistical significance for low and medium N inputs (see Figure 2), it is possible that other nutrients—such as phosphorus (P) and potassium (K)—contributed to plant biomass variation. These nutrients have also been shown to be released by microbiome predators through predation on the microbiome (Bonkowski 2004; Gebremikael et al. 2016). While biomass increased, chlorophyll as well as soil (MBC, MBN, C, N, C/N ratio) and plant nutrient content (leaf C, leaf N, leaf S and leaf C/N ratio) were not affected by microbiome predator addition. These results contradict our first hypothesis, but are supported by research showing that an increase in plant biomass due to protist addition is not necessarily linked to an increase in nutrient content in the shoots (Ekelund et al. 2009). This apparent contradiction may be explained by the ability of microbiome predators to release essential nutrients beyond N and C—such as P and potassium (K) (Gebremikael et al. 2016; Mei et al. 2025), thereby contributing to more balanced plant nutrition. Apart from a more balanced plant nutrition, microbiome predators can also release certain growth hormones either directly or via changing the microbiome composition, increasing plant biomass without affecting the relative nutrient concentrations (Bonkowski 2004). In our setup, it was also evident that microbiome predator addition shaped fungal communities. While this result is not expected to come from direct predation by nematodes, as the ones used in our study were obligate bacterivores, protists were previously suggested to modulate fungal communities mainly indirectly via changes in bacterial composition but also as potential generalist feeders (Berlinches de Gea et al. 2024; Geisen 2016; Huang et al. 2021). In contrast, N addition had a moderately positive effect on biomass compared to the stronger effects of predator diversity, but notably improved soil and plant nutrient content. This aligns with studies indicating that excess N is stored in the plant without affecting biomass when other nutrients (P, K) are a limiting factor instead of N (Bonkowski 2004; Gebremikael et al. 2016).

### 4.2 Microbiome Predator Diversity on Plant Performance

Most importantly, we here show how an increasing diversity of microbiome predators led to a plant biomass increase, in line with our second hypothesis. While we acknowledge that the communities inoculated in the experiments most probably changed throughout the experiment, the consistent effects observed on different soil and plant biochemical parameters, specifically increasing plant biomass and modifying bacterial composition, suggest that the inoculation was successful, explained by diversity level‐specific modulation of bacterial communities.

This microbiome predator diversity‐induced increase in plant biomass aligns with the BEF concept, which states a positive relationship between diversity and ecosystem functioning (Tilman et al. 2014). This concept has been largely tested and documented aboveground, with plant diversity positively affecting plant productivity in famous experimental setups such as Cedar Creek or the Jena experiment (e.g., Dietrich et al. 2023; Tilman and Downing 1994), but barely experimentally tested with soil biodiversity (Berlinches de Gea, Li, et al. 2023). The main explanation behind the positive relationship is thought to be the complementarity of niches (time, space, feeding habits, etc.) among different species. The niche complementarity in our setup is likely derived from species‐specific predation differences among nematode and protist species (Amacker et al. 2022; Berlinches de Gea et al. 2024; Hu et al. 2024), broadening the potential feeding niches with increasing species richness of predators. In this way, more nutrients are thought to be released and taken up by the plant (Gao et al. 2019). This hypothesis is further explained by the notable reduction of soil fungi when increasing microbiome predator diversity. This reinforces the idea that sharing the same function (here microbiome predation) does not imply functional redundancy but complementarity (Eisenhauer et al. 2023). However, we show that the N content in the soil did not change with increasing microbiome predator diversity, indicating that broader feeding niches might have enhanced the release of other nutrients like K or P, as previously reported (Bonkowski 2004; Gebremikael et al. 2016).

When the N input level was modeled as a continuous variable, we did not detect a statistically significant interaction between microbiome predator diversity and N input levels on plant biomass (*p* = 0.61). However, when N was modeled as a categorical factor, a significant interaction emerged (Figure S4). This indicates that the effects of microbiome predator diversity differ among the individual nitrogen input levels, but not in a linear manner across the N gradient. We therefore interpret these differences as non‐linear, level‐specific responses, rather than a continuous interaction effect. These effects were observed in pots without N addition and under low and intermediate N levels, but not under high N conditions (Figure S4). This pattern could be explained by Liebig's law of the minimum, which states that plant growth is constrained by the most limiting resource (von Liebig 1840). Thus, at high N input levels, where nitrogen was not limiting, the influence of microbiome predator diversity diminished.

### 4.3 Microbiome Predator Diversity Shaped Plant Biomass via Bacterial Community Composition

Partially in contrast with our third hypothesis, increasing microbiome predator diversity was correlated with total plant biomass and bacterial community composition but not with soil nutrients or other soil and plant parameters. Importantly, bacterial community composition emerged as the strongest predictor of plant biomass, linking predator diversity to plant performance and suggesting feeding preferences of microbiome predators (Amacker et al. 2022; Berlinches de Gea et al. 2024; Liu et al. 2017). Thus, increasing microbiome predator diversity enhanced the complementarity of feeding niches. These targeted feeding patterns are often associated with increases in plant growth‐promoting bacteria or reductions in plant pathogenic bacteria, ultimately contributing to an increase in plant biomass (Asiloglu et al. 2020; Guo et al. 2022; Ingham et al. 1985; Jiang et al. 2020; Krome et al. 2009). In our case, while we showed that certain plant‐beneficial genera such as *Burkholderia* (reported as plant growth‐promoting (Pal et al. 2022)) or *Kaistia* (reported to suppress the fungal plant pathogen *Fusarium oxysporum* (Cazzaniga et al. 2023)) were enhanced in treatments with increasing microbiome predators, changes in the bacterial functional profile are of more importance. For example, N cycling functions like “nitrogen respiration” and “ureolysis” declined with increasing microbiome predator diversity, suggesting potential shifts in N cycling pathways such as reduced N losses to the environment and greater N retention for plant use. C cycling was also likely enhanced as “aerobic chemoheterotrophic” bacteria, which are linked to the formation of organic matter in soils (Labouyrie et al. 2023), increased with an increasing protist diversity. Our results thus support the fact that the microbial loop involves more processes than N release, as shown by Bonkowski (2004). Additionally, we reveal profound changes in the functional profile of bacterial communities induced by predation.

Increasing N input levels, rather than microbiome predator diversity, had an impact on chlorophyll and plant nutrient content parameters, which is in line with other studies showing N‐dependency responses of leaf nutrient contents (Firn et al. 2019). This plant nutrient N‐dependency was shown to be independent of microbiome predator diversity, indicating that the benefits from enhancing microbiome predator diversity existed regardless of the N input levels and vice versa. Similarly, N input levels, rather than microbiome predator diversity, shaped the majority of the soil biochemical properties, suggesting that nutrient availability directly regulates microbial activity, whereas predator diversity influences microbial community structure. This reinforces the idea of the complementary role that N and microbiome predator diversity have. Finally, the effects of microbiome predator diversity on the microbial and auxiliary loops also applied when N was added. Consequently, the sBEF concept could be used as a framework for targeted agricultural treatments unlike some other approaches, which only work in low nutrient conditions.

## 5 Conclusions

In this study, we show that microbiome predator diversity is a key driver of plant performance. By experimentally manipulating microbiome predator diversity, we demonstrate that increasing diversity of microbiome predators enhances plant biomass most likely via changes in the soil bacterial community composition. Furthermore, the data presented here recognize microbiome predators as potential biostimulants as previously suggested (Hu et al. 2023), and add empirical evidence linking microbiome predator diversity to plant performance within the context of microbial nutrient cycling (i.e., microbial loop theory). These findings suggest that the application or stimulation of diverse microbiome predator communities may provide greater benefits than single‐species inoculants.

Our results further show that N addition and microbiome predator diversity may play different and complementary roles in plant performance: increasing N levels affected chlorophyll and plant nutrient content, while microbiome predator diversity mainly affected plant biomass. Our findings show that microbiome predator diversity indirectly enhances plant biomass via changes in the soil bacterial community composition as well as by promoting nutrient release, probably beyond only N. Importantly, our experiment was intended as a controlled model system rather than a direct representation of field conditions. Thus, we emphasize the need to further test the observed sBEF relationships in different soils, such as agricultural soils varying in available nutrients or soil compaction.

Overall, our results emphasize that the diversity of microbiome predators, rather than their mere presence, plays a central role in shaping soil microbial communities and their consequences for plant growth. Explicitly accounting for microbiome predator diversity in agricultural sBEF frameworks may therefore improve our understanding of how soil food webs regulate plant productivity and nutrient dynamics.

## Author Contributions

**Joshua Both:** conceptualization, investigation, methodology, validation, writing – review and editing, data curation. **Nina Haas:** conceptualization, investigation, methodology, validation, writing – review and editing, data curation. **Florian Wichern:** conceptualization, methodology, writing – review and editing, supervision, project administration, validation. **Rutger A. Wilschut:** writing – review and editing, formal analysis, methodology. **Stefan Geisen:** project administration, conceptualization, writing – review and editing, methodology, supervision, investigation, validation. **Alejandro Berlinches de Gea:** conceptualization, investigation, writing – original draft, methodology, validation, visualization, writing – review and editing, formal analysis, data curation, supervision.

## Conflicts of Interest

The authors declare no conflicts of interest.

## Supporting information


**Figure S1:** Experimental design detailing the components of each treatment. On top, the background microbiome inoculated to all the pots and the four levels of biodiversity are depicted. All treatments were replicated 10 times. The pots containing 0 protist (P) and 0 nematode (Nem) species under no N regime were used as a general control for all the treatments while pots with 0 protist, 0 nematodes, and N regimes were used as a control within treatments.
**Figure S2:** Microbiome predator addition increases both shoot (A) and root (B) but not the root/shoot ratio (C), irrespective of diversity levels (X‐axis shared for all panels). Small dots represent the raw data, error bars represent the standard deviation, and the thicker dot represents the mean. Statistically significant effects (ANOVA; *p* < 0.05) are shown with an asterisk.
**Figure S3:** Microbiome predator addition, irrespective of diversity levels, shape fungal abundance depending on the amount of N added. Small dots represent the raw data, error bars represent the standard deviation, and thicker dots represent the mean.
**Figure S4:** The relationship between plant total biomass (g) and increasing diversity of microbiome predators is dependent on the different N input levels (A–D). Small dots represent the raw data, error bars represent the standard deviation, and the thicker dot represents the mean. The test results shown in the panels are based on the individual models performed for each N level. The X‐axis is shared per column.
**Figure S5:** Relationship between Shannon alpha diversity index with microbiome predator (A) and nitrogen addition (B). Small dots represent the raw data, error bars represent the standard deviation and the thicker dot represents the mean.
**Figure S6:** Relationship between Shannon (A–D) and chao1 (E–H) alpha diversity indexes varies with an increasing microbiome predator addition (X‐axis) per level of N input. The test results shown above the figure are based on the linear mixed‐effect models performed to test the interaction between increasing diversity and N input levels. The nitrogen levels are written above each subplot.
**Figure S7:** Differential abundance analysis of bacteria at the genus level. Treatments compared are microbiome predator addition versus non‐addition. Red bars on top and blue bars on the bottom represent statistically significant genera with a Log2 fold change higher than 2 and lower than −2, respectively. Genera with values > −2 and < 2 were removed for readability purposes. The results should be seen as follows: positive Log2 fold change values: those genera increased when microbiome predators were added. Negative Log2 fold change values: those genera decreased when microbiome predators were added.
**Figure S8:** Differential abundance analysis of bacteria at the genus level. Treatments compared are shown on top of each graph. From left to right: 6 versus 0 species, 12 versus 0 species, 24 versus 0 species, 12 versus 6 species, 24 versus 6 species and 24 versus 12 species. Red bars on top and blue bars on the bottom represent statistically significant genera with a Log2 fold change higher than 2 and lower than −2, respectively. These results must be understood as genera that either increased or decreased in relative abundance when comparing the first treatment against the second.
**Figure S9:** Correlation plot among biotic and abiotic parameters measured. Empty spots represent no statistical significance (Pearson corr; *p* > 0.05), while coloured ones represent statistically significant correlations (Pearson corr; *p* < 0.05). False Discovery Rate (FDR) correction was applied for *p* values using the Benjamini‐Hochberg procedure. The size of the circle depicts the degree of significance (the bigger, the smaller the value for P) and the color refers to the direction of the correlation (brown ‐or left side of the bar‐ is negatively correlated, green ‐or right side of the bar‐ is positively correlated). The icons refer to the nature of the variable (Treatment, plant biomass, leaf, microbiome, or soil‐related). MP, Microbiome Predator.

## Data Availability

Raw sequence data obtained through amplicon sequencing are available at the NCBI Sequence Read Archive (SRA) database under the Bioproject PRJNA1493190. Collected data supporting this study are available in a Zenodo repository (https://doi.org/10.5281/zenodo.21336195), including sample metadata, plant biomass, soil physicochemical, and soil biological data.
